# The cost of first-ever stroke in Valle d’Aosta, Italy: linking clinical registries and administrative data

**DOI:** 10.1186/1472-6963-12-372

**Published:** 2012-10-30

**Authors:** Edo Bottacchi, Giovanni Corso, Piera Tosi, Massimo Veronese Morosini, Giuseppe De Filippis, Laura Santoni, Gianluca Furneri, Cristina Negrini

**Affiliations:** 1 Department of Neurology, Regional Hospital of Aosta Valley, Aosta, Italy; 2 Department of Statistics, Regional Hospital of Aosta Valley, Aosta, Italy; 3 Healthcare Direction, AUSL of Aosta Valley, Aosta, Italy; 4 Department of Outcome Research Pfizer, Rome, Italy; 5 Scientific Direction, Italian National Research Center on Aging (I.N.R.C.A.), Ancona, Italy; 6 Life Science Division, Simon-Kucher & Partners, Milan, Italy

**Keywords:** Stroke, Costs, Italy, Prevention, Administrative claims, Record linkage

## Abstract

**Background:**

Stroke is one of the most relevant reasons of death and disability worldwide. Many cost of illness studies have been performed to evaluate direct and indirect costs of ischaemic stroke, especially within the first year after the acute episode, using different methodologies.

**Methods:**

We conducted a longitudinal, retrospective, bottom-up cost of illness study, to evaluate clinical and economic outcomes of a cohort of patients affected by a first cerebrovascular event, including subjects with ischaemic, haemorrhagic or transient episodes. The analysis intended to detect direct costs, within 1, 2 and 3 years from the index event. Clinical patient data collected in regional disease registry were integrated and linked to regional administrative databases to perform the analysis.

**Results:**

The analysis of costs within the first year from the index event included 800 patients. The majority of patients (71.5%) were affected by ischaemic stroke. Overall, per patient costs were €7,079. Overall costs significantly differ according to the type of stroke, with costs for haemorrhagic stroke and ischaemic stroke amounting to €9,044 and €7,289. Hospital costs, including inpatient rehabilitation, were driver of expenditure, accounting for 89.5% of total costs. The multiple regression model showed that sex, level of physical disability and level of neurological deficit predict direct healthcare costs within 1 year. The analysis at 2 and 3 years (per patient costs: €7,901 and €8,874, respectively) showed that majority of costs are concentrated in the first months after the acute event.

**Conclusions:**

This cost analysis highlights the importance to set up significant prevention programs to reduce the economic burden of stroke, which is mostly attributable to hospital and inpatient rehabilitation costs immediately after the acute episode. Although some limitation typical of retrospective analyses the approach of linking clinical and administrative database is a power tool to obtain useful information for healthcare planning.

## Background

The traditional definition of a stroke, [1], is “a neurological deficit of cerebrovascular cause that persists beyond 24 hours or is interrupted by death within 24 hours”. Worldwide, stroke is one of the most common causes of death, long-term morbidity and disability [2-4]. Annually, 15 million people worldwide suffer a stroke [5]. Of these, 5 million die and another 5 million are left permanently disabled, placing a burden on family and community. Moreover, due to the progressive population ageing, the absolute number of stroke incidence is expected to increase. By 2030, stroke would be the leading cause of 10.4% and 11.8% of male and female annual deaths, respectively [6].

Owing to the high level of morbidity associated with stroke, the economic burden of this disease is substantial. For 2008, it was estimated that stroke would cost the US economy $ 65.5 billion in healthcare services, medications, and lost productivity [7].

Stroke can be classified into two major categories: ischaemic and haemorrhagic. Ischaemic stroke is caused by an interruption of the blood supply, while haemorrhagic stroke results from a rupture of a blood vessel or an abnormal vascular structure. 87% of strokes are caused by ischemia and the remainder by haemorrhage [8].

Many international [9-19] and national [20,21] studies have estimated healthcare costs and resource utilization associated with ischaemic stroke, particularly those costs and hospitalizations that arise from non-stroke-related cardiovascular events in the post-stroke follow-up period. Conversely, there is still poor information on the economic burden of haemorrhagic stroke.

Cost-of-illness (COI) analysis is the main method of providing an overall view on the economic impact of a disease [22]. In Italy, two different studies have evaluated the economic burden of stroke in Italy, using various approaches. Gerzeli et al., in the ECLIPSE study [20], evaluated direct and indirect costs of stroke using a longitudinal, incidence-based methodology. Morsanutto et al. [21] estimated cost and outcomes after first stroke hospital admission using administrative databases.

Region Valle d’Aosta owns a large informative system on administrative patients’ claims, collecting records on healthcare services offered to the resident population (prescriptions, ambulatory interventions, hospitalizations). In the same timeframe the Regional Valle d’Aosta Hospital has set up a stroke clinical registry, including patients who have been hospitalized since 1 January 2004 to date. The main purpose of the present study is to link the two clinical and administrative sources in order to: 1) estimate the current burden of stroke in Valle d’Aosta; 2) evaluate the risk of death and of further hospitalization due to cardio-cerebrovascular reason; 3) evaluate the annual costs of stroke comparing the costs according to the type of cerebrovascular event; 4) assess the contribute of each cost component on total annual costs; 5) determine the relation between costs, demographic and clinical status of patients with stroke.

## Methods

### Study design

**Figure 1:**
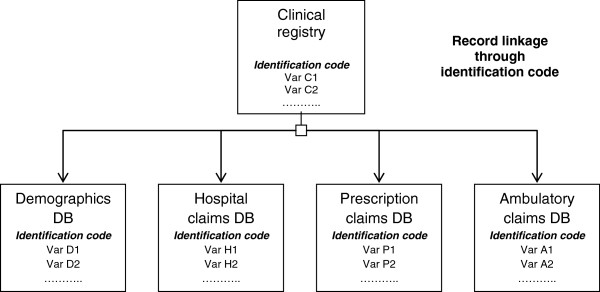
Structure of database linkage*.

The present study is a longitudinal, retrospective, bottom-up cost of illness study, aimed to evaluate clinical and economic outcomes of a cohort of patients affected by a first cerebrovascular event (stroke or transient ischaemic attack, TIA). The analysis is conducted in the perspective of the Italian regional healthcare system: only direct costs have been evaluated. In the main analysis, patients who were hospitalized for a cerebrovascular accident, included in the disease clinical registry, matching inclusion criteria, were observed up to 1 year after hospital admission or death, whichever came first. The date of hospital admission was the index date and coincided with the beginning of observation. Dates of death and/or next hospitalizations due to cardio-cerebrovascular event, if applicable, were detected through record linkage between the clinical registry, the demographic database and the hospitalization database, respectively. Economic resources used by patients were detected through record linkage between regional administrative healthcare archives and the clinical registry (Figure 1). Cerebrovascular-related resource consumption data on hospitalizations, pharmaceutical prescriptions, ambulatory interventions (also including patients’ rehabilitation), after index date were collected. Additional analyses were performed to assess clinical outcomes and costs over a period of two and three years.

### Data sources

Data from different sources were integrated through database linkage and then used to conduct the analysis: 1) data included in the clinical disease registry; 2) administrative data on demographic status of the Val d’Aosta resident population; 3) data on pharmaceutical prescriptions; 4) data on ambulatory and specialistic visits; 5) data on hospitalizations. Data from datasets 1, 2, 4 and 5 covered the period between January 1, 2004 and December 31, 2007. Data from dataset 3 covered the period between January 1, 2003 and December 31, 2007.

As sensitive patients’ information was stored in data sources, the present study was submitted to the Ethical Committee of Aosta LHU, which approved it. Also, in order to assure patients’ privacy, identification codes used in both administrative databases and clinical registries were preliminary transformed into alpha-numeric codes, so that researches could not have access to sensitive data.

### Inclusion and exclusion criteria

To be included in the analysis, subjects must be recorded in the disease clinical registry, collecting all hospital admissions due to cerebrovascular disease (confirmed stroke or TIA) occurred in the period January 1, 2004, December 31, 2007. The following subjects were excluded: 1) subjects with invalid demographic status at the index date (resulting as not resident or emigrated); 2) subjects who were not present in the database of hospital records (invalid database linkage); 3) subjects aged < 18 years; 4) subjects with previous documented stroke; 4) subjects not potentially observable for 1 year (index date after January 1, 2007). Patients with history ischemic transient attack (TIA) were not excluded in the analysis, as it was considered a risk factor for major cerebrovascular events (as well as other conditions like myocardial infarction, atrial fibrillation, etc.). Patients who, at the last day of observation, were alive but did not reach 1 year of follow-up, were excluded from the analysis in order to avoid overestimation of annual costs (complete follow-up period is not possible, and patients could generally use higher amount of resources during first months after disease onset).

### Clinical and economic outcomes estimation

The demographic and clinical characteristics of the sample population were obtained from the clinical registry, which collected detailed information of social status, job, clinical and pharmacological anamnesis, status of the disease at hospital admission. In particular, Barthel Index [23] and NIHSS (National Institute of Health Stroke Scale) [24] scores were collected to estimate the level of physical dependency and the level of neurological deficit, respectively.

Survival analysis was conducted using the Kaplan-Meier method, to estimate time to death and time to fatal or non-fatal cardio-cerebrovascular event for the sample population, over the follow period. In the analysis of time to fatal and non-fatal event, a patient was supposed to have a failure if resulted either dead or if he had hospital admission with at least one of the following ICD-9 [25] (International Statistical Classification of Diseases and Related Health Problems, 9^th^ Revision) diagnoses: 43X.XX (cerebrovascular diseases), 342.XX (hemiplegia and hemiparesis), 410.XX, 411.XX, 413.XX, 414.XX (myocardial infarction, angina pectoris, other ischaemic diseases), 424.XX, 426.XX, 427.XX, 428.XX (myocardial diseases, conduction disturbances, arrhythmia and heart failure), 785.XX, 780.0X, 784.3X, 784.5X (alterations and disturbances of the nervous system, attributable to cerebrovascular accident). Patients were censored either if emigrated (or temporary transferred) within the observation period, or at the end of the observation period.

Economic resources consumed during the observation period were collected from regional administrative databases. For each of the patients recorded in the disease clinical registry, healthcare interventions occurred within the observation period were collected. We collected economic resources which were directly attributable to the cerebrovascular event: 1) drugs used for cardio-cerebrovascular prevention (anti-thrombotic agents, anti-diabetes agents, anti-hypertension agents, lipid-lowering drugs; 2) hospitalizations due to cardio-cerebrovascular events (according to the above mentioned classification); 3) routine ambulatory procedures for stroke patients, defined by investigators, including rehabilitation costs.

Two different cost of illness indicators were calculated: 1) the average yearly patient cost (regardless of observation time, which could be <365 days if the patient died before the end of the observation period), and 2) the cost per patient per year, which was calculated by dividing the overall amount of costs (sum of patient costs) during the year after the index date by the overall time of observation (sum of patient observation times). In addition, the average contribute of each cost component was evaluated.

Finally, two different analyses were performed to test cost differences among homogenous groups of patients. In the first analysis, we compare resource consumptions over by different forms of cerebrovascular diseases (ischaemic stroke, haemorrhagic stroke, undetermined stroke, TIA), level of physical disability and neurological deficit. In the second analysis, we evaluated the relation between overall costs and age, sex, type of stroke, level of disability, level of neurological deficit, previous cardiovascular disease and observation time (analysis of predictors).

### Statistical analysis

Analyses of socio-demographic status of the sample, clinical conditions prior to the index event, level of disease severity at hospital admission, as well as survival analyses were purely descriptive. As regards the cost analyses, two different approaches were adopted. Univariate (parametric and non-parametric) analysis was used to compare annual costs by stroke type, physical disability, neurological deficit, while Generalized Linear Model (GLM) with log link and inverse Gaussian family was used to study the relation between costs and independent variables. This methodological approach has been extensively used in predicting costs in recent [26-29].

## Results

### Analysis at 1 year

Overall, 800 (58.0%) of 1,380 patients included in the registry were included in the analysis. Reasons of exclusion are shown in Table 1. 272 patients (19.7%) having a previous diagnosis of major cerebrovascular disease, were excluded from the sample. Tables 2 and 3 summarize the main demographic and clinical characteristics of the sample, respectively. The mean age was 74.9 years (SD: ±13.5) and the sample was equally distributed between males and females (48.6% and 51.4%, respectively). The majority of patients (n=572, 71.5% of overall sample) was affected by confirmed ischaemic stroke, while haemorrhagic stroke occurred in 96 cases (12.0%). 19 cases (2.4%), for whom it was not possible to clearly determine the form of stroke, were defined as “undetermined”. 88 (11.0%) patients were previously affected by myocardial infarction. The most frequent risk factor among the sample population was hypertension, diagnosed in 76.4% of patients prior to index date. 141 (17.6%) and 202 (25.3%) subjects were also affected by diabetes and dyslipidemia, respectively. The average length of stay of the hospitalization determining the cerebrovascular event was 16.6 days (SD:±18.2).

**Table 1:** Reasons for exclusion criteria

Exclusion criteria*	**No**. **of patients excluded**	**(%)**
Index date not allowing a 365 days - observation period	362	(45.3%)
Previous major cerebrovascular event	272	(34.0%)
Venous trombo-embolism	1	(<1.0%)
Age < 18 years	1	(<1.0%)

* Patients can have more than one exclusion criteria at the same time.

**Table 2:** Sample characteristics: demographic variables

**Variable**	800	(100%)
**Total patients** – **No** (%)		
Male	389	(48.6%)
Female	411	(51.4%)
< 45 years	32	(4.0%)
45–64 years	120	(15.0%)
65–74 years	176	(22.0%)
75–84 years	309	(38.6%)
85 years	163	(20.4%)
**Age**, **years** – Mean (± SD)	74.9	(±13.5)
Total number of patients	795	(100%)
Housewife	22	(2.8%)
Not occupied	1	(<1.0%)
Retired	675	(84.0%)
Employed	97	(12.2%)
Total number of patients	795	(100%)
Lives in community	39	(4.9%)
Lives alone	194	(24.4%)
Lives with others (family)	562	(70.7%)

**Table 3:** Sample characteristics: clinical variables


**Total patients** – **No** (%)	800	(100%)
**Type of stroke** – **No** (%)		
Ischaemic	572	(71.5%)
Haemorrhagic	96	(12.0%)
TIA (Transient ischaemic attack)	113	(14.1%)
Undetermined	19	(2.4%)
**Comorbidities and risk factors** – **No** (%)		
Previous myocardial infarction	88	(11.0%)
Diabetes	141	(17.6%)
Hypertension	611	(76.4%)
Dyslipidemia	202	(25.3%)
Smoke	124	(15.5%)
Familiarity for CV diseases	77	(9.6%)
Previous TIA	53	(6.6%)
Atrial fibrillation	129	(16.1%)
Heart failure	56	(7.0%)
**Body mass index** (**Kg**/**m**^**2**^)		
**No** (%)	592	(100.0%)
Mean (± SD)	25.1	(3.8)
**Level of disability measured with Barthel Index at admission**		
**No** (%)	790	(100.0%)
Totally independent (0–20)	133	(16.8%)
Minimally dependent (21–60)	8	(1.0%)
Moderately dependent (61–90)	229	(29.0%)
Severely dependent (91–99)	184	(23.3%)
Totally dependent (100)	236	(29.9%)
**Level of neurological deficit measured with National Institutes of Health Stroke Scale** (**NIHSS**) **at admission**		
**No** (%)	790	(100.0%)
No neurological deficit (NIHSS=0)	83	(10.5%)
Light neurological deficit (NIHSS=1-7)	449	(56.8%)
Moderate neurological deficit (NIHSS=8-14)	110	(13.9%)
Severe neurological deficit (NIHSS ≥15)	148	(18.7%)

**Figure 2:**
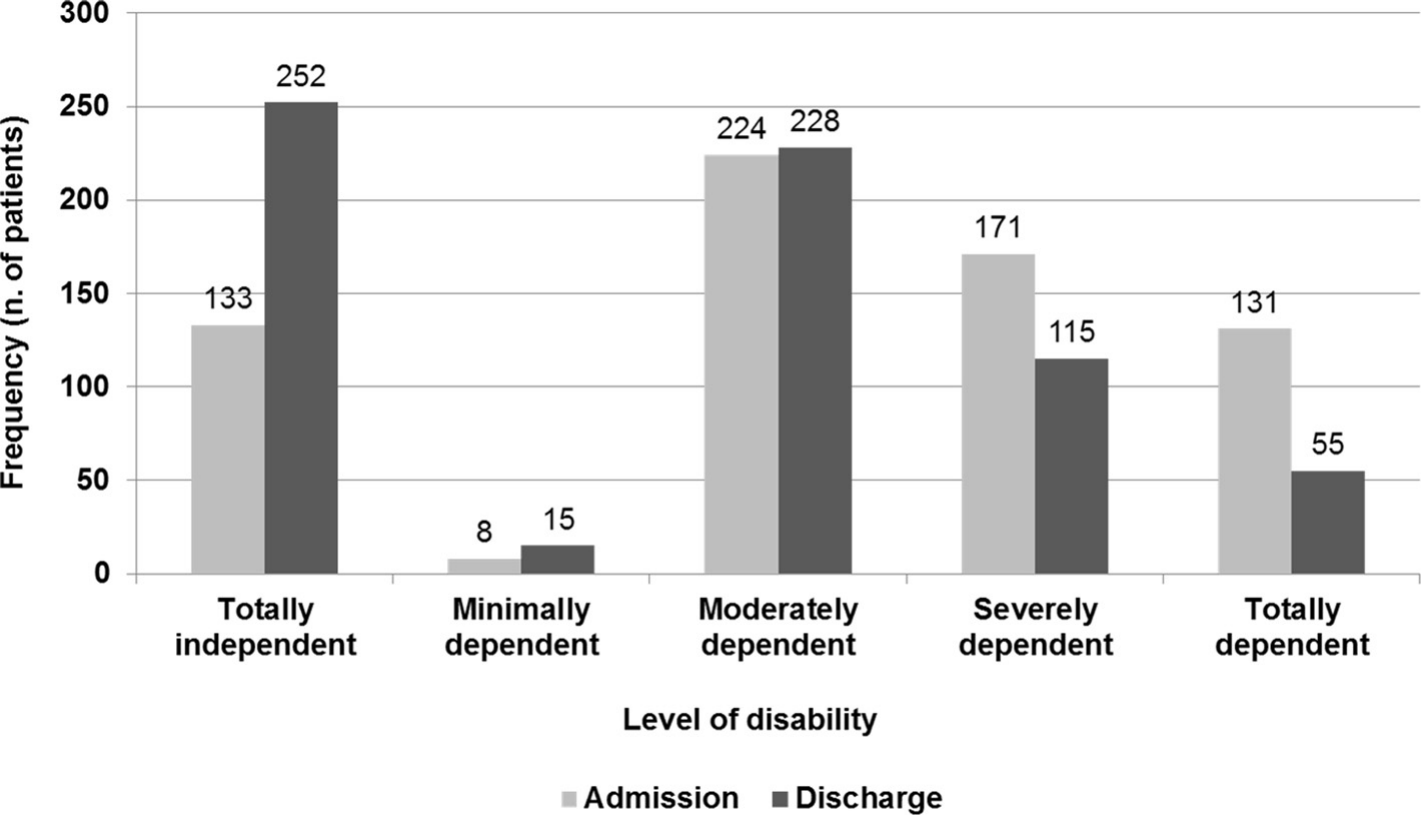
Distribution of patients according to Barthel index at hospital admission and discharge (n=667).

**Figure 3:**
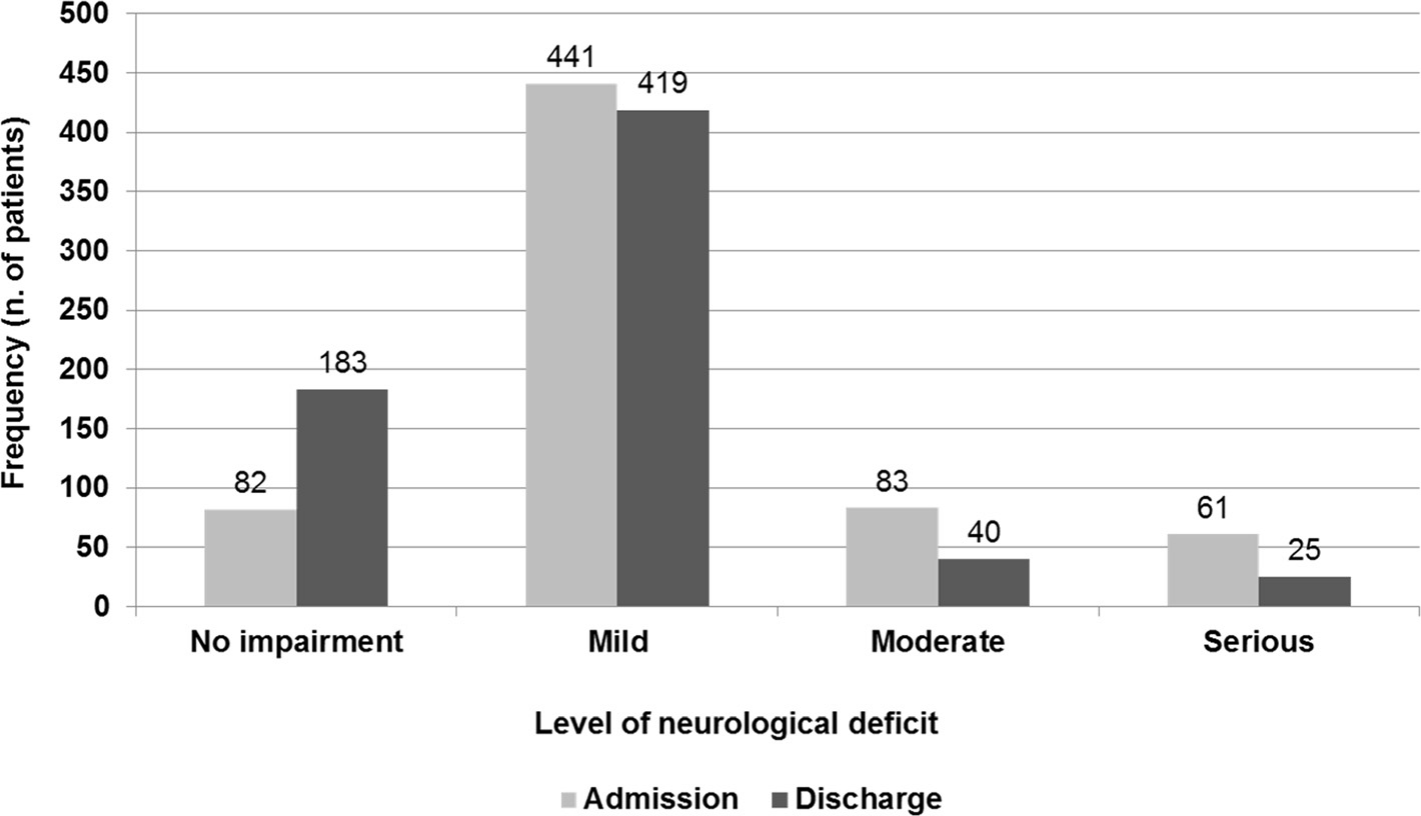
Distribution of patients according to NIHSS scores at hospital admission and discharge (n=667).

At admission, 53.2% of patients showed a moderate to severe level of disability, measured with Barthel Index (score between 91 and 100). However, in those 667 subjects with Barthel index measured both at admission and at discharge, a significant reduction of the disability level was observed (Figure 2, chi-square test, p<0.001). As regards the neurological deficit, 32.6% of patients showed a moderate to serious level of disability. However, similarly to physical disability, a significant improvement of neurological status was observed at the end of hospitalization (Figure 3, chi-square test, p<0.001).

**Figure 4:**
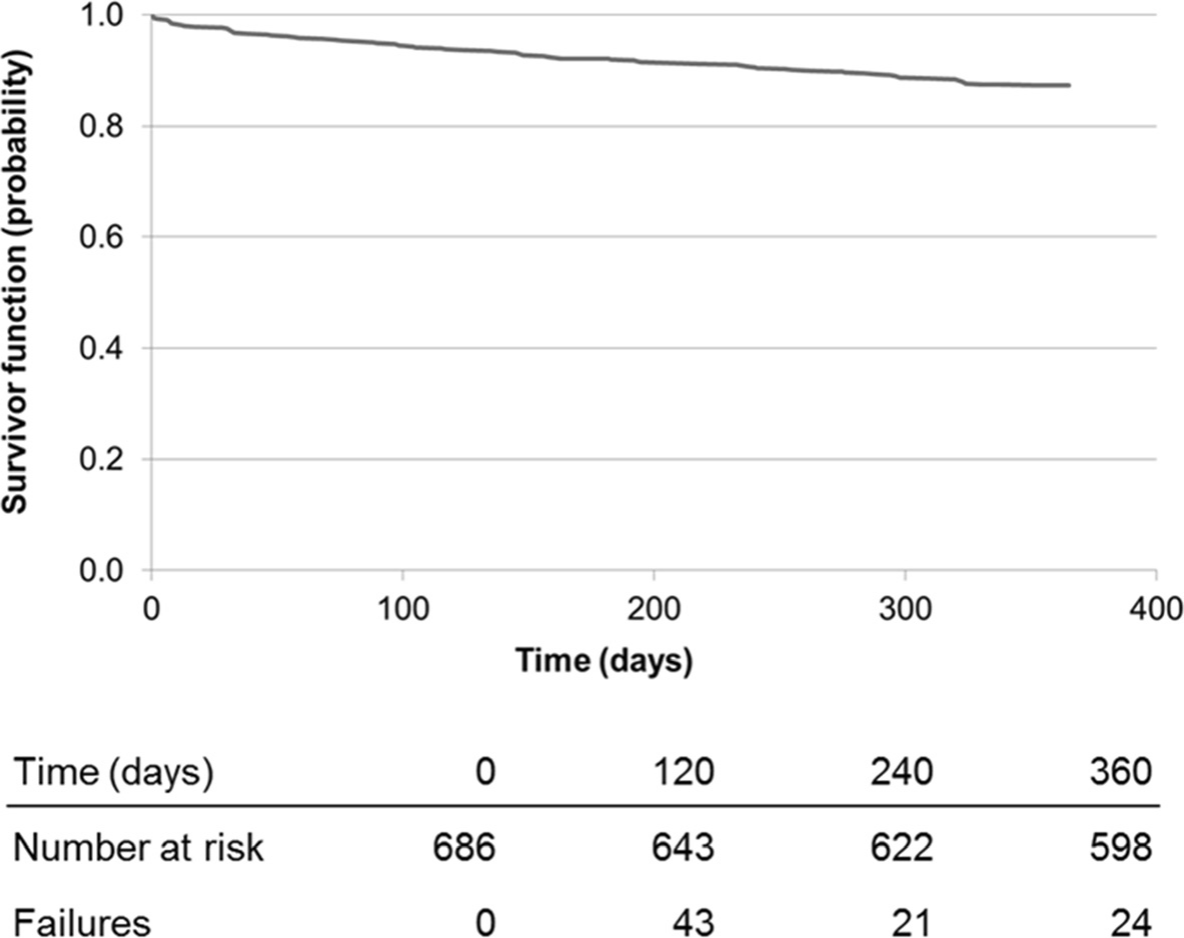
Kaplan-Meier survival analysis to determine time to death.

**Figure 5:**
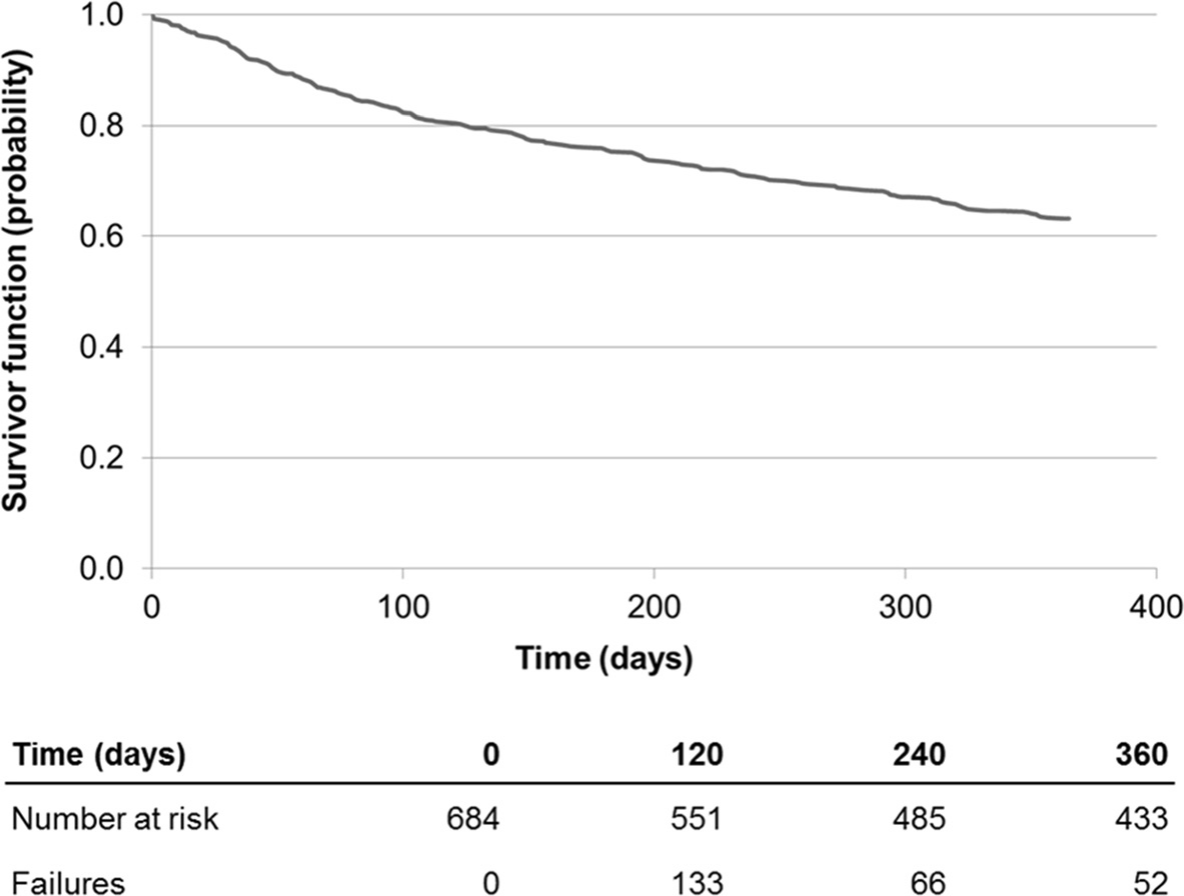
Kaplan-Meier survival analysis to determine time to death or fatal or non-fatal cardio-cerebrovascular event.

Of the 800 patients, 114 (14.3%) died during hospital staying, while other 88 (11.0%) died within the first year after index date. Overall, about 13 deaths per 100 patients per year occurred. Figure 4 shows survival function for the 686 patients who were discharged alive after cerebrovascular event. In this cohort, survival probabilities were 92.1% and 87.2%, at 180 days and 360 days after the index date. Figure 5 shows the Kaplan-Meier estimation of time to fatal or non-fatal cardio-cerebrovascular events. In the first 365 days following the index date, 252 of 684 subjects included in this analysis (36.8%) died or had cardio-cerebrovascular event. The risk of death or non-fatal cardio-cerebrovascular accident in the first 6 months after the index date was approximately 25%.

After 1 year of observation, the overall cost per patient was €7,079. Distribution of average costs per patients was asymmetric, with a median value of €4,580. During the first year of observation, the overall healthcare expenditure was €5,663,259. This expenditure was generated in 231,013 overall days of follow up (about 289 days per patient observed), resulting in €8,498 per patient per year of observation (approximately €24.5 per patient per day). Table 4 shows the cost composition from discharge to year of follow-up. Hospital costs (€6,340) represent the main driver of expenditure, accounting for 89.5% of the total direct costs (these costs also include inpatient rehabilitation costs, which could not be separated from management costs for the acute event). Costs for outpatient rehabilitation were 40.1% of the ambulatory costs (total: €164 per patient). The average per patient cost was sensibly different according to survival time: as expected, patients surviving ≥ 30 days (n=669) costs €7,531, respect to patients surviving <30 days or dead during hospital staying (n=131; €4,770). As shown in Table 5, the frequency of anti-thrombotic agents, anti-hypertension drugs was above 75% among patients surviving ≥30 days after the index date. In the same group, almost all patients (96.3%) had at least an access for ambulatory services (diagnostic tests, laboratory analysis, physician visits, etc.).

**Table 4:** Composition of average, per patient annual costs

**Type of costs**		
**Total costs**, € (% **on total costs**)	**7**,**079**	(**100**%)
**Hospital costs**, € (% **on total costs**)	**6**,**340**	(**89**.**5**%)
**Ambulatory costs**, € (% **on total costs**)	**409**	(**5**.**8**%)
Specialistic visits	72	(17.6%)
Instrumental tests	72	(17.6%)
Laboratory analyses	99	(24.2%)
Rehabilitation	164	(40.1%)
Other	2	(0.5%)
**Drug costs**, € (% **on total costs**)	**330**	(**4**.**7**%)
Anti-thrombotic drugs	43	(13.0%)
Anti-hypertension drugs	208	(63.0%)
Anti-diabetes drugs	26	(7.9%)
Lipid-lowering drugs	53	(16.1%)

**Table 5:** Frequency of resource usage in the ≥30 days survivors (n=669)


Hospitalization – No (%)	669	(100%)
Ambulatory care	644	(89.5%)
Anti-thrombotic drugs	516	(77.1%)
Anti-hypertension drugs	531	(79.4%)
Anti-diabetes drugs	100	(14.9%)
Lipid-lowering drugs	165	(24.7%)

**Figure 6:**
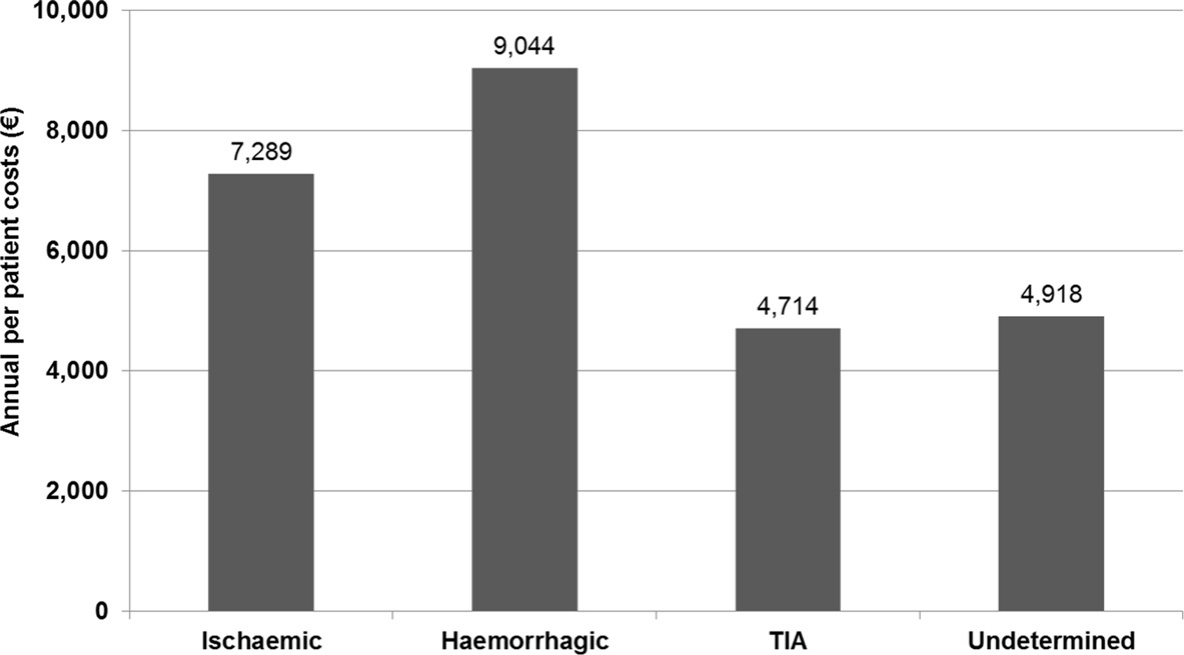
Average annual costs, by type of stroke.

Analysis of variance in univariate analyses showed a statistically significant difference (p<0.0001) of costs by type of stroke (Figure 6). This trend was also confirmed through non-parametric tests Fischer tests, evaluating the different distribution of cost quartiles among the groups (p<0.0001). Similar statistical results were achieved analyzing costs by level of physical disability at hospital discharge (totally independent: €5,192.4; minimally dependent: €5,108.9; moderately dependent: €7,306.9; severely dependent: €10,461.5; totally dependent: €12,702.4) and level of neurological deficit (no neurological deficit: €4,926.7; light neurological deficit: €7,327.3; moderate neurological deficit: €15,546.7; severe neurological deficit: €15,204.9).

Finally, a generalized linear regression was elaborated to study the relation between costs (dependent variable), demographic and clinical variables (independent variables). The log-link model successfully met diagnostic tests (Pearson correlation test: 0.6277, Pregibon link test: 0.7963, modified Hosmer and Lemeshow: 0.8811; p>0.05 values allowing analysis requirements). The modified-Park test suggested the usage of the inverse Gaussian family function (according to which variance is proportional to the cube of the mean). As shown in Table 6, the generalized model indicates that sex, level of neurological deficit (measured with NIHSS) and level of disability (measured with Barthel index) affected overall annual costs. Unlike univariate analysis, type of stroke was not found to influence annual costs. Other demographic and clinical variables (age, previous myocardial infarction, length of follow up) did not significantly predict cost variability. Generally, patients with moderate and severe neurologic impairment cost 105% and 96% more than patients without neurologic impairment, and patients with moderate and high level of disability (severely or totally dependent) cost 27% and 52% more than patients without physical disability.

**Table 6:** Results of the generalized linear model analysis

**Variables**	**Exp(b)**			**P value**
		**Lower limit**	**Upper limit**	
Female	Reference			
Male	1.174949	1.057331	1.305651	0.003
First quartile (20.3-68.1 yrs)	Reference			
Second quartile (68.3- 77.5 yrs)	1.016946	0.882974	1.171244	0.816
Third quartile (77.5-83.9 yrs)	0.987269	0.851009	1.145346	0.866
Fourth quartile (83.9-98.7 yrs)	0.850884	0.718406	1.007793	0.061
Ischemic stroke	Reference			
Hemorrhagic stroke	1.009120	0.813787	1.251339	0.934
TIA	0.877714	0.741751	1.038599	0.129
Undetermined	0.812306	0.548259	1.203519	0.300
No neurological deficit	Reference	0.991601	1.356929	0.064
Light neurological deficit	1.159971			
Moderate neurological deficit	2.047076	1.390864	3.012889	0.000
Severe neurological deficit	1.960430	1.236072	3.109273	0.004
Totally independent	Reference			
Minimally dependent	0.909896	0.661837	1.250929	0.561
Moderately dependent	1.270401	1.096781	1.471505	0.001
Severely or totally dependent	1.519167	1.259055	1.833018	0.000
No	Reference			
Yes	1.087153	0.903138	1.308661	0.377
**Observation time** (**days**)	0.999009	0.997970	1.000050	0.062

Number of observations = 667.

Log likelihood = −9281.635433.

AIC* = 27.87897.

BIC** = −4233.279.

* Akaike information criterion.

** Bayesian information criterion.

### Analysis at 2 and 3 years

**Figure 7:**
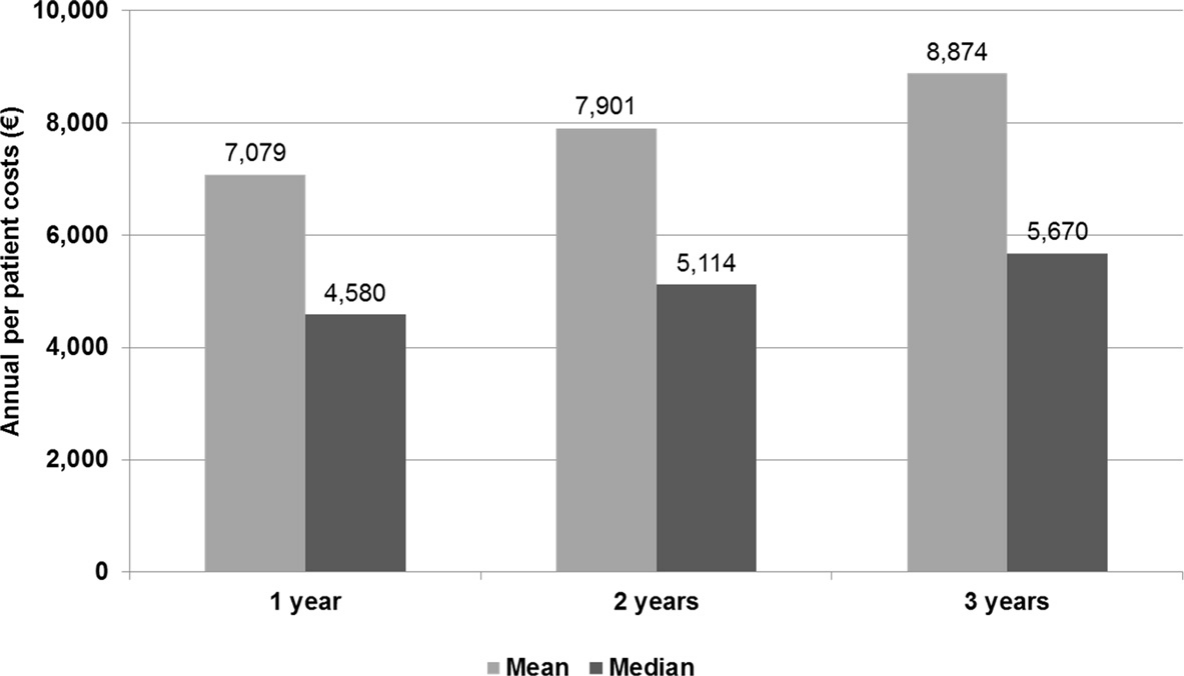
Per patient costs, at 1, 2 and 3 years from index date.

The analysis at 2 years included 549 patients. 655 of the initial 1,380 (47.1%) subjects were excluded from the analysis since they could not be observed for at least 2 years (index date prior to January 1, 2006). Most demographic (age, sex) and clinical characteristics of the sample (distribution by type of stroke, Barthel Index, NIHSS scores) were not different from the sample observed in 1-year analysis. The overall death risk at the end of the second year of observation was 21%, while the risk of death or non-fatal cardio-cerebrovascular event for the same period was 47%. The overall per patient cost in the first two years from index date was €7,901 (median: €5,114, Figure 7), with hospital costs remaining the main cost driver, accounting for 84.8% of total costs.

Subjects eligible for the analysis at 3 years were 268. As well as in the analysis at 3 years, demographic and clinical average values and distribution remain quite similar. Kaplan Meier survival analysis showed a risk of death or non-fatal cardio-cerebrovascular accident of 29% and 59% at the end of year 3 from index date, respectively. The overall costs during the 3 years amounted to €8,874 (median: €5,670, Figure 7).

## Discussion and conclusion

The present cost of illness analysis is an example of integration of two informative sources (clinical and administrative data) providing complementary information. Many cost of illness studies [30-34] have been conducted using administrative claims. Although suitable as approach for evaluating direct healthcare costs, pure administrative database analysis shows some limitations. First, such analyses cannot capture indirect costs (i.e. loss of productivity), therefore are not suitable to conduct cost of illness analyses in the society perspective. Second, characterization of sample population is often poor and based on assumptions. Both clinical and socio-demographic information (i.e. severity of the disease, level of disability, comorbidities, job, social status, etc.) are not available and this makes studies from administrative databases hardly usable for inferential analysis. On the other hand, perspective, observational economic studies are designed to clearly match objectives defined in the study protocol. However, longitudinal, perspective studies are time consuming and their implementation requires large economic. In many cases, data collection is made through questionnaires which are directly administered to the patients. This approach is not always reliable and could generate mistakes and incompleteness of results.

The approach used in the present study intends to overcome the typical drawbacks of administrative database analyses. The integration with a clinical registry allows a detailed characterization of the patient at the index date. The inclusion of incident patients assures homogeneous evaluation of costs, and the characterization type of cerebrovascular event at baseline permits to obtain information on forms such as haemorrhagic stroke, which has been less frequently evaluated by health economists. Demographic and clinical characterization was also useful to run cost prediction models. Statistical results (level of correlation, adjusted R-squared) show similar findings of the model elaborated by Gerzeli et. al [20] in their longitudinal analysis.

Cost of stroke within the first year of occurrence is concerning. A patient affected by first stroke episode costs, on average, €24.5 per day. Observed daily costs are increasingly higher for increasing level of physical disability and level of neurological deficit. Observed costs for haemorrhagic events were the highest among different forms of stroke, although this variable did not explain costs variability in the regression model. Direct costs for haemorrhagic stroke patients in the first year after the event were quite low if compared with those of other countries. Dodel et al., in their analysis of subarachnoid hemorrhage in Germany reported an annual direct cost of €22,482, most of which (92%) covered by health insurance [35]. Cost composition and evolution of overall management costs at 1, 2, 3 years suggest two important aspects. First, inpatient management and rehabilitation represent the main cost driver (about 90% of direct costs at year 1). Post-event healthcare interventions (pharmacological treatment and follow up visits) do not seem requiring significant amount of resources. Second, the comparison between costs at years 1, and costs at year 2 and 3, clearly suggested that most of the healthcare expenditure is concentrated in the first months after the acute event. This aspect highlights the enormous importance, for our healthcare service, to invest more in prevention. In particular, life-style modifications and pharmacological interventions on selected, at high-risk populations should be intensified. In our analysis, we retrospectively observed the usage of certain drugs preventing CV events in our sample population. As example, only 41% and 15% of the sample received an anti-thrombotic therapy and lipid-lowering treatment before the index date, respectively.

Previous experiences of stroke cost of illness studies showed a certain variability of results, maybe due to the different methodological approaches adopted. However, costs estimated in the present study (€8,498 per patient per year of observation), however are lower, but somehow comparable to previous findings (Gerzeli et al. [20], €6,111 for direct healthcare costs in the first 180 days after stoke), especially considering that: 1) our sample also included patients with TIA, consuming less resources than patients with stroke; 2) distribution of costs is not homogeneous over the first year, with most resources consumed during the first months after the acute episode; 3) determination of attributable costs is subject to expert judgment and therefore can differ across studies. Moreover, other international studies found similar trends for long term care of stroke patients [9]. The cost difference between cardio-embolic stroke and TIA was also found by Winter et al., in their recent work [36]. The same authors also highlight the acute stroke care as the most relevant cost contributor in management of stroke patients.

The present study shows some limitation regarding the assessment of rehabilitation costs, which could not be break-downed from hospital costs through our informative sources. Therefore these costs were included in the analyses, but we were not able to determine their impact on the overall healthcare expenditure. The analysis of SDO [37] (Hospital Discharge Forms) highlights that LOS (length of stay) of certain patients was longer than 2 weeks, suggesting that those patients were likely moved from the neurology ward to the rehabilitation ward to initiate physiotherapy programs . However, we did not have additional information to estimate the amount of days spent in each of the two wards. The second limitation is due to the choice of including only costs being directly attributable to the cerebrovascular event with high level of evidence. As example, we included in the cost measurements only post-event hospitalizations for cardio-cerebrovascular events and evaluated drug expenditure for therapies used in cardio-cerebrovascular prevention. Direct costs could have been slightly underestimated; however we preferred to maintain this conservative approach rather than including costs attributable to other underlying causes. Third, this analysis does not capture indirect costs, which are relevant in stroke [35], but for which a different study approach (mainly based on perspective data) should be adopted.

Our analysis, focused on Region Valle d’Aosta, does not provide any cost comparison among Italian regions. Further research should be oriented to replicate and improve this methodological approach and compare costs by geographical area. A recent study from Frolich et al. [38] highlights large difference of costs when different healthcare systems are compared to each other. In this case, cost differences may be also attributed to different methodological approach. However, we have reason to believe that minor differences would exist among Italian regions, due to a quite standardized hospital care management and a similar access to medical therapies in the different regions.

In conclusion, integrating clinical and administrative data, whereas applicable, seems extremely powerful to obtain reliable and useful information to support evidence-based healthcare programs. Moreover, it permits to retrospectively analyze data in a fast and not expensive way. We believe that this approach, if further refined, will be increasingly exploited by our policy makers in the future.

## Competing interests

The study was sponsored by Pfizer Italy. Simon-Kucher and Partners received grant from Pfizer Italy to participate in the study, contribute to the analysis and draft the manuscript. EB, GC, PT, MVM, GDF declare they have no competing interests and have not received grants or funding to participate in the study. At the time of study conduction, GF was a full-time employee of Simon-Kucher and Partners and received a salary from Simon-Kucher and Partners. GF was previously a full employee of Pfizer Italy (May 2004-October 2008). At the time of study conduction, LS was a full-time employee of Pfizer Italy and received a salary from Pfizer Italy. CN is a full-time employee of Simon-Kucher and Partners and receives a salary from Simon-Kucher and Partners.

## Authors’ contributions

EB, GC, PT, LS, GF and CN participated in the study design and approved the final version of the present article. MVM managed data extraction, data cleaning and record linkage of the different databases used in the analysis. MVM and GF performed statistical analysis. EB, GC, PT, LS and CN supervised statistical analysis. All authors contributed to interpretation of analysis results. EB, GC, PT and GF wrote the manuscript for scientific publication. LS, GDF, CN reviewed the final content of the manuscript. EB, GC, LS and GF coordinated the overall analysis project. All authors read and approved the final manuscript.

## Pre-publication history

The pre-publication history for this paper can be accessed here:

http://www.biomedcentral.com/1472-6963/12/372/prepub
